# Surveillance of HIV-1 *pol* transmitted drug resistance in acutely and recently infected antiretroviral drug-naïve persons in rural western Kenya

**DOI:** 10.1371/journal.pone.0171124

**Published:** 2017-02-08

**Authors:** Harris Onywera, David Maman, Seth Inzaule, Erick Auma, Kennedy Were, Harrison Fredrick, Prestone Owiti, Valarie Opollo, Jean-François Etard, Irene Mukui, Andrea A. Kim, Clement Zeh

**Affiliations:** 1 Center for Global Health Research (CGHR), Kenya Medical Research Institute (KEMRI), Kisumu, Kenya; 2 Epicentre, Médecins Sans Frontières (MSF), Paris, France; 3 TransVIHMI IRD UMI 233 –INSERM U 1175 –Université de Montpellier, Montpellier, France; 4 National AIDS and STI Control Programme (NASCOP), Ministry of Health, Nairobi, Kenya; 5 US Centers for Disease Control and Prevention (CDC), Nairobi, Kenya; 6 US Centers for Disease Control and Prevention (CDC), Kisumu, Kenya; Universidad Autonoma de Madrid Centro de Biologia Molecular Severo Ochoa, SPAIN

## Abstract

HIV-1 transmitted drug resistance (TDR) is of increasing public health concern in sub-Saharan Africa with the rollout of antiretroviral (ARV) therapy. Such data are, however, limited in Kenya, where HIV-1 drug resistance testing is not routinely performed. From a population-based household survey conducted between September and November 2012 in rural western Kenya, we retrospectively assessed HIV-1 TDR baseline rates, its determinants, and genetic diversity among drug-naïve persons aged 15–59 years with acute HIV-1 infections (AHI) and recent HIV-1 infections (RHI) as determined by nucleic acid amplification test and both Limiting Antigen and BioRad avidity immunoassays, respectively. HIV-1 *pol* sequences were scored for drug resistance mutations using Stanford HIVdb and WHO 2009 mutation guidelines. HIV-1 subtyping was computed in MEGA6. Eighty seven (93.5%) of the eligible samples were successfully sequenced. Of these, 8 had at least one TDR mutation, resulting in a TDR prevalence of 9.2% (95% CI 4.7–17.1). No TDR was observed among persons with AHI (n = 7). TDR prevalence was 4.6% (95% CI 1.8–11.2) for nucleoside reverse transcriptase inhibitors (NRTIs), 6.9% (95% CI 3.2–14.2) for non- nucleoside reverse transcriptase inhibitors (NNRTIs), and 1.2% (95% CI 0.2–6.2) for protease inhibitors. Three (3.4% 95% CI 0.8–10.1) persons had dual-class NRTI/NNRTI resistance. Predominant TDR mutations in the reverse transcriptase included K103N/S (4.6%) and M184V (2.3%); only M46I/L (1.1%) occurred in the protease. All the eight persons were predicted to have different grades of resistance to the ARV regimens, ranging from potential low-level to high-level resistance. HIV-1 subtype distribution was heterogeneous: A (57.5%), C (6.9%), D (21.8%), G (2.3%), and circulating recombinant forms (11.5%). Only low CD4 count was associated with TDR (p = 0.0145). Our findings warrant the need for enhanced HIV-1 TDR monitoring in order to inform on population-based therapeutic guidelines and public health interventions.

## Introduction

Highly active antiretroviral therapy (HAART) has been effective at treating HIV infection and improving overall health and survival, but constant viral evolution continues to result in drug resistance [1]. Of the 28.3 million HIV-infected persons eligible for antiretroviral therapy (ART) in resource-limited settings (RLS), only 34% are on ART under the 2012 World Health Organization (WHO) treatment guidelines [2]. Based on the 2013 Kenya AIDS Indicator Survey, 58% of people living with HIV/AIDS (PLWHA) aged 15–64 years in Kenya were eligible for ART, but only 63% of them were found to have been initiated [3]. Primary, or transmitted drug resistance (TDR) [4] has been of a rising concern in sub-Saharan Africa (sSA) with scale-up and long-term use of antiretrovirals (ARVs) [5–7]. It accounts for 8–22% among newly HIV-infected persons in the majority of the regions of the world [8–13]. TDR may complicate the management of PLWHA [14,15], and as proposed by Hassan et al. and Nichols et al., it may reverse the benefits made from global scale-up of ART [16,17].

As access to HAART is rolled out globally, WHO Global HIV Resistance Network recommends periodic monitoring of TDR (among acutely and recently infected drug-naïve persons [mean seroconversion period: 180 days [13,18]]) in RLS [19,20] where there is limited availability and treatment options of ARVs, hence ensuring effective treatment [10,21,22]. TDR has a potential to compromise treatment [17,22–24] despite apposite prescribing and adherence [15]. In resource-rich settings (RRS), moderate levels of TDR have been observed [4] but are either stabilizing or declining due to universal availability of highly efficacious drugs [25]. Many regions in sSA have low to moderate TDR levels, but urban sites have begun to show an increase [6,10,11,16,26–33]. Data on TDR in RLS are scarce, with drug resistance testing not routinely performed [21].

WHO recommends that TDR should be assessed periodically among newly infected persons who are identified using a set of criteria that is likely to select persons who have AHI/RHI. These include drug-naïve young (<25 years of age) and newly HIV diagnosed primagravida women visiting antenatal clinics, persons visiting voluntary counselling and testing/sexually transmitted infections clinics, or in high-risk populations, although limitations exist [8,13]. The use of these criteria is limiting as most of the incident cases are omitted from the survey [20]. Some studies in RRS evaluate TDR among persons with AHI, but this method is difficult to perform, and takes time to attain a sufficient number of persons [8,13]. In addition to routine monitoring of HIV prevalence, WHO recommends evaluating HIV incidence in high prevalence settings through population-based surveys by laboratory-based assays for AHI/RHI [18,34]. Cross-sectional incidence surveys are not dependent on patient follow-up and rapidly offer up-to-date information that is required for efficient public health responses [35]. Moreover, such surveys may additionally be used to determine TDR.

Characterizing HIV drug resistance using the AHI/RHI method is likely to give a more representative estimate [10,19,34,36]. We assessed HIV-1 TDR level in treatment-naïve persons with AHI/RHI in an ARV-exposed rural setting in western Kenya that has both high HIV incidence and prevalence [37,38].

## Materials and methods

### Study design and population

The TDR survey was nested within the Ndhiwa HIV Impact in Population Survey (NHIPS) [37], an incidence study conducted in Ndhiwa sub-county of Homa Bay County in Nyanza region of western Kenya, between September and November 2012. A multistage cluster sampling method was used to recruit study participants aged 15–59 years from 3,302 randomly selected households. Those who volunteered to participate in the study were interviewed and tested for HIV using a serial rapid testing algorithm. CD4 cell count, recency of HIV infection, and viral load for those who tested HIV positive were measured using validated laboratory procedures. Of the 6,076 persons eligible for NHIPS, 1,457 (24.1%) were confirmed as HIV-1 infected [37]. We collected the baseline characteristics of HIV-1 infected persons from the NHIPS records, and drug-naïve cases with AHI/RHI infection (n = 93) were included in the study.

### Ethical considerations

The NHIPS was approved by the Ethics Committee of the Kenya Medical Research Institute (KEMRI), Kenya; the Committee for the Protection of Persons, Saint-Germain-en-Laye, France; and the Division of HIV/AIDS Prevention (DHAP) under the Centers for Disease Control and Prevention (CDC), Atlanta, US. All the participants provided written informed consent to be involved in the study. For participants younger than 18 years of age but older than 15 years of age and not a mature minor, the study details were explained to at least one parent/guardian for parental written permission and then to the minor for written consent. Mature minors are defined in the Kenya National voluntary counselling and testing guidelines as persons under 18 who are married, pregnant, parents, engage in behaviour that put them at HIV transmission risk or are child sex workers. However, mature minors were strongly advised to speak with and involve their parents/guardians in the study participation process if they felt it was safe to do so. All minors, unless matured, had parental/guardian written permission and also provided written consent.

### HIV status and duration of recency

HIV status was determined using a parallel testing algorithm of two HIV rapid tests: Uni-Gold^TM^ HIV (Trinity Biotech, Ireland) and Determine^TM^ HIV-1/2 (Abbott Laboratories^®^, Tokyo, Japan). The recency of HIV infection was established by two serological assays: the Limiting Antigen (LAg) (Maxim Biomedical, Inc., Rockville, USA) and BioRad Avidity Index (AI) (Bio-Rad Laboratories, WA, USA) enzyme immunoassays (EIA). Assay cut-offs for the duration of recency were 1.5 normalized optical density (ODn) and 30% AI, respectively, for the two serological assays. Any sample that was identified as recently infected from either assay, was included in the study. Persons with AHI were identified by negative result with Determine^TM^ (Abbott Laboratories^®^, Tokyo, Japan) and positive result with nucleic acid amplification test (NAAT) [37]. CD4 cell count and viral load were measured using PIMA point-of-care CD4 Analyzer (Alere, Inc., Waltham, MA, USA) and COBAS AmpliPrep/COBAS TaqMan v2.0 (Roche Molecular Systems, Branchburg, NJ, USA), respectively, according to the manufacturers’ instructions.

### Genotypic drug resistance profiling

Dried blood spots (DBS) and plasma from persons with AHI and RHI, respectively, were used for HIV-1 genotyping. The partial *pol* gene (protease, PR: codons 6–99; reverse transcriptase, RT: codons 1–251) of the HIV-1 isolates was sequenced using a CDC-validated in-house genotyping assay utilizing a standard sequencing chemistry as previously described [39]. This was performed on ABI PRISM 3130*xl* Genetic Analyzer (Applied Biosystems, Foster City, CA, USA). Sequence chromatograms were analyzed by RECall v2.0 Software using HIV-1 HXB2 sequence as the reference [40], and checked for cross-contamination by phylogenetic analyses (www.phylogeny.fr/) [41]. TDR was defined according to the Stanford HIVdb’s (hivdb.stanford.edu/) Calibrated Population Resistance (CPR) v6.0, and WHO 2009 Mutation List [42]. We employed the WHO TDR surveillance classification scale for prevalence: low (<5%), intermediate/moderate (5–15%) and high (>15%) [20]. The predicted ARV drug responses in persons with TDR was assessed by the Stanford HIVdb v7.0 that is normalized to five drug response levels based on net drug score: Susceptible (≤9), Potential low-level resistance (10–14), Low-level resistance (15–30), Intermediate resistance (31–59), and High-level resistance (≥60) [43].

### Phylogenetic analyses

HIV-1 subtyping was statistically done by maximum likelihood (ML) phylogenetic reconstruction in MEGA6; a bootstrap of 1000 replicates was used to generate the consensus evolutionary tree and assess the strength of cluster from each node. Nineteen known reference sequences from Los Alamos National Laboratory HIV sequence database (http://hiv-web.lanl.gov) of common HIV-1 subtypes in Kenya were included to deduce the subtypes of the ingroup. HIV-1 subtype K was used as an outgroup species. Multiple sequence alignment was achieved by MUSCLE program within MEGA6. The alignment was trimmed to a length of 1035 bp. According to the Bayesian Information Criterion scores of 24 different nucleotide substitution models, the Hasegawa-Kishino-Yano with discrete Gamma distribution (0.42) (for non-uniformity of evolutionary rates among sites: 5 rate categories) and Invariable sites (0.54) (HKY+G+I) was selected as the ideal model to describe the substitution pattern. All positions containing gaps or missing data were completely eliminated. Subtree-Pruning-Regrafting–Extensive (SPR level 5) method was used as the ML heuristic option for tree inference due to its effectiveness. The initial tree was automatically generated by BioNJ. A moderate branch swap filter was set in order to allow for a semi-stringent exhaustive optimization of the branch lengths and improvements in log likelihood values [44].

### Statistical analyses

The overall TDR and drug-specific TDR prevalence were assessed in accordance with WHO standards [20] and the 95% CI computed based on Wilson’s approach [45]. Associations of TDR with categorical (gender, recency of HIV-1 infection, and HIV-1 subtype) and continuous (participant’s age, CD4 cell count, and viral load) variables were computed by Fisher’s exact and Wilcoxon signed-rank tests, respectively. The overall TDR prevalence and patterns were descriptively compared to previous surveys in East Africa.

## Results

### Population baseline characteristics

Of the 6,076 eligible persons, 1,457 (24.1% after adjustment) were confirmed as HIV-1 infected, of which 93 (6.4%) were either acutely (n = 7) or recently-infected (n = 86) and ARV drug-naïve by self-report. None of the persons included in the survey reported being aware of or exposed to prophylaxis for prevention of mother-to-child transmission (PMTCT). Table 1 shows the key baseline characteristics of the 93 persons with AHI/RHI included in the analysis.

**Table 1:** Demographic and clinical characteristics of acutely and recently HIV-1 infected antiretroviral-naïve persons in the Ndhiwa cohort, Kenya, 2012.

Baseline Characteristics		All Persons (n = 93)
Duration of Infection	Recently Infected	86 (92.0%)
	Acutely Infected	7 (8.0%)
Median Age (years)		29 (IQR 23–37)
Gender	Male	26 (28%)
	Female	67 (72%)
Median CD4^+^ T-Cell Count (cells/μl)		550 (IQR 387–698)
Viral Load (copies/ml)	Recently Infected	4.68 log_10_ (IQR 4.13–5.21)
	Acutely Infected	6.18 log_10_ (IQR 5.89–7.00)

IQR—Interquartile Range.

The median age was 29 years (interquartile range (IQR) 24–37), and 72% (67) were female. The median CD4 cell count and viral load were 550 cells/μL (IQR 387–698) and 4.68 log_10_ copies/mL (IQR 4.13–5.21), respectively. Median viral load for persons with AHI was 6.18 log_10_ copies/mL (IQR 5.89–7.00).

### Drug resistance patterns

Our genotyping success rate was 94% (n = 87). Six (6%) samples (including two DBS) failed to amplify due to inadequate sample volume, poor sample integrity, or low viral load and were therefore excluded from the analyses. Eight of the genotyped samples had at least one TDR mutation (TDRM) resulting in an overall TDR prevalence of 9.2% (95% CI 4.7–17.1). The distribution of class-specific TDRMs in the eight persons with TDR was quantified. Five (62.5%) had only one any class-specific TDRM (i.e., 1 person with PI TDRM, 1 with NRTI TDRM, and 3 with NNRTI TDRMs) while the rest had dual-class NRTI/NNRTI resistance. Class-specific TDR prevalence was 4.6% (95% CI 1.8–11.2) for NRTIs, 6.9% (95% CI 3.2–14.2) for NNRTIs, and 1.2% (95% CI 0.2–6.2) for PIs. In total, 10 TDRMs were observed: one in the protease (PR) gene and 9 in the reverse transcriptase (RT) gene. The dominant TDRMs in the RT gene were K103N/S (n = 4, 4.6%) and M184V (n = 2, 2.3%), while in the PR gene only M46I/L (n = 1, 1.1%) occurred. We found no TDR among persons with AHI.

### TDR prevalence and pattern comparison

We further compared the observed TDR with those observed in other regions of East Africa (Table 2).

**Table 2:** Prevalence of TDR in selected regions of East Africa.

Country	Region	TDR Prevalence (%)	Year(s) of Assessment	HIV-1 TDR Eligibility among ARV-Naïve Persons
**Kenya**	Nairobi	7.5	2005	HIV-infected persons aged ≥18 years who presented themselves for treatment at a clinic. No history of ARV use by self-report [32].
**Kenya**	Nairobi	4.5	2007/09	HIV-infected persons aged ≥18 years, eligible to start first-line ARV therapy in accordance with the treatment guidelines. All pregnant women were excluded [27].
**Kenya**	Mombasa	4.9	2007/09	HIV-infected persons aged ≥18 years, eligible to start first-line ARV therapy in accordance with the treatment guidelines. All pregnant women were excluded [27].
**Kenya**	Mombasa	13.2	2009/10	Newly diagnosed HIV-infected persons aged 18–25 years, or laboratory evidence of HIV recency (defined by a positive antibody test with a negative antibody test in the past 1 year, or an indeterminate/negative antibody test with detectable HIV RNA or p24 antigen) [26].
**Kenya**	Kilifi	1.1	2008/10	Cross-sectional pretreatment HIVDR assessment from HIV-infected persons aged >15 years [16].
**Kenya**	**Ndhiwa**	**9.2**	**2012**	See our **Methods** section: Subsections–Study design and population, and HIV Status [37] and duration of recency.
**Tanzania**	Dar es Salaam	<5.0	2005/06	HIV-infected primagravidas aged <25 years in the HIV sentinel surveillance among pregnant women attending antenatal clinic [28].
**Uganda**	Kampala	7.0	2002/04	HIV-infected persons from a HIV RNA monitoring study. No history of ARV use by self-report [29].
**Uganda**	Entebbe	0.0	2006/07	HIV-infected primagravidas aged 13–22 years with no previous positive HIV test, and ≥500 CD4 cell count (cells/mL) [33].
**Uganda**	Entebbe	19.2	2006/09	Incident cases: Persons that seroconverted during 1- or 3-month. Persons (including women in antenatal care) not followed in the HIV incidence studies but had documentations of a previous HIV-negative test results were also eligible. HIV recency defined by a positive antibody test with a negative antibody test in the past 1 year, or an indeterminate/negative antibody test with detectable HIV RNA or p24 antigen) [10].
**Uganda**	Mbarara	3.0	2005/10	HIV-infected persons from a HIV RNA monitoring study. No history of ARV use by self-report [29].
**Uganda**	Southwestern Uganda	1.4	2004/10	Incident cases: Persons that seroconverted during 3-month follow-up for 6 years and their CD4 cell count results were available. Estimated date of seroconversion was the midpoint between the last seronegative (antibody-negative test result) date and the first seropositive (antibody-positive test result) date [31].
**Uganda**	Kampala	8.6	2009/10	Newly diagnosed persons aged 18–25 years voluntary counseling and testing centers, or laboratory evidence of HIV recency (defined by a positive antibody test with a negative antibody test in the past 1 year, or an indeterminate/negative antibody test with detectable HIV RNA or p24 antigen) [11].
**Uganda**	Masaka, Wakiso, Mukono	6.4	2009/11	Incident cases: Persons with high-risk behaviour who seroconverted during 6-month follow-up for 2 years. Estimated date of seroconversion was the midpoint between the last seronegative date and the first seropositive date [30].

Except for Entebbe and Mombasa (current updates), the observed TDR was relatively higher than those of selected regions within East Africa, published over the last decade [11,16,26–32]. All the class-specific TDRMs were higher than those reported in rural parts of East Africa [16,30,31]. Whereas only NRTI TDRM was the highest when compared with TDR data in Mombasa [26], the contrast was true when compared with Kampala [29].

### Clinical significance of observed TDRMs

**Fig 1:**
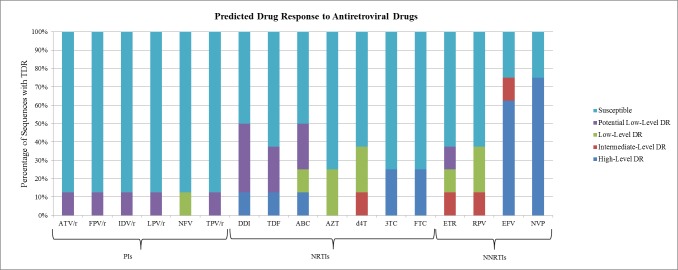
Predicted antiretroviral (ARV) drug responses of eight persons with TDR mutations. Drug responses were based on the Stanford HIVdb v7.0 report. The y-axis indicates the percentage number of sequences with TDR while the x-axis indicates the different ARV drug classes that were affected by the TDR mutations. Drugs that were unaffected by any particular TDR were excluded from the analyses. TDR—Transmitted Drug Resistance; PIs—Protease Inhibitors; NRTIs—Nucleoside Reverse Transcriptase Inhibitors; NNRTIs—Non-Nucleoside Reverse Transcriptase Inhibitors; ATV/r—Boosted Atazanavir; FPV/r—Boosted Fosamprenavir; IDV/r—Boosted Indinavir; LPV/r—Boosted Lopinavir; NFV—Nelfinavir; TPV/r—Boosted Tipranavir; r—Ritonavir; 3TC—Lamivudine; ABC—Abacavir; AZT—Zidovudine; D4T—Stavudine; DDI—Didanosine; FTC—Emtricitabine; TDF—Tenofovir; EFV—Efavirenz; ETR—Etravirine; NVP—Nevirapine; RPV—Rilpivirine.

According to the Stanford HIVdb v7.0, predicted resistance to the first-line drugs among the patients with TDR was 75% intermediate- to high-level resistance to efavirenz and nevirapine (recommended first-line NNRTI drugs), 25% to the cytosine analogues; lamivudine and emtricitabine and about 13% to tenofovir, abacavir, stavudine and didanosine (recommended NRTI drugs), and rilpivirine and etravirine (NNRTI drugs). None of the patients had intermediate- to high-level resistance to zidovudine or PI drugs (Table 3 and Fig 1).

**Table 3:** ARV drug resistance levels among persons with TDR.

Patient ID	TDR Mutation(s)	Other Non-Polymorphism(s)	Drugs Affected: Stanford HIVdb Drug Interpretation		
			PIs	NRTIs	NNRTIs
**0210801**	M184V, G190A	K101H	-	DDI^a^, ABC^b^, 3TC^d^, FTC^d^	EFV^d^, ETR^b^, NVP^d^, RPV^b^
**0301602**	K103N	-	-	-	EFV^d^, NVP^d^
**0631702**	M46IL	-	ATV/r^a^, FPV/r^a^, IDV/r^a^, LPV/r^a^, TPV^a^, NVP^b^	-	-
**0921502**	K103N	V11I	-	-	EFV^d^, NVP^d^
**1170802**	K65R, M184V, K103S, V106M	L10IL, A62V	-	D4T^c^, 3TC^d^, ABC^d^, DDI^d^, FTC^d^, TDF^d^	EFV^d^, NVP^d^
**1201401**	K103N	-	-	-	EFV^d^, NVP^d^
**1211302**	L210W	E138A	-	ABC^a^, TDF^a^, DDI^a^, AZT^b^, D4T^b^	ETR^a^, RPV^b^
**1311802**	Y181C, K219N	V75I, V90I	-	ABC^a^, DDI^a^, TDF^a^, AZT^b^, D4T^b^	EFV^c^, ETR^c^, RPV^c^, NVP^d^

PI—Protease Inhibitors; NRTIs—Nucleoside Reverse Transcriptase Inhibitors; NNRTIs—Non-Nucleoside Reverse Transcriptase Inhibitors; ATV/r—Boosted Atazanavir; FPV/r—Boosted Fosamprenavir; IDV/r—Boosted Indinavir; LPV/r—Boosted Lopinavir; NFV—Nelfinavir; TPV/r—Boosted Tipranavir; r—Ritonavir; 3TC—Lamivudine; ABC—Abacavir; AZT—Zidovudine; D4T—Stavudine; DDI—Didanosine; FTC—Emtricitabine; TDF—Tenofovir; EFV—Efavirenz; ETR—Etravirine; NVP—Nevirapine; RPV–Rilpivirine.

a = Potential low-level resistance (mutation net drug score (10–14).

b = Low-level resistance (mutation net drug score of 15–30).

c = Intermediate resistance (mutation net drug score of 31–59).

d = High-level resistance (mutation net drug score of ≥ 60).

Hyphen (-) denotes that none of the ARV drugs was affected.

### HIV genetic diversity

**Fig 2:**
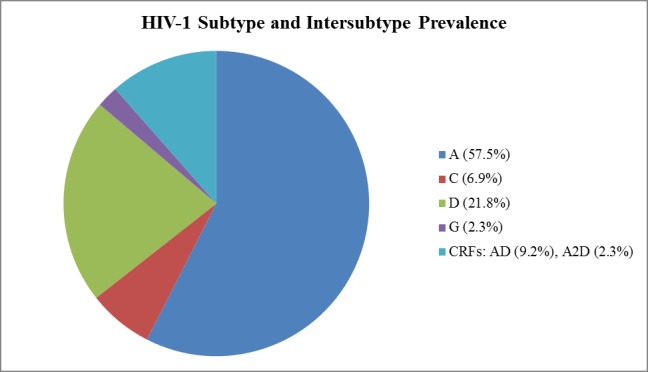
Summary of the subtyping and intersubtyping results of the phylogenetic analyses represented by pie chart.

HIV-1 subtype was inferred from the consensus evolutionary tree (S1 Fig). The subtype distribution was heterogeneous with subtype A predominating (n = 50, 57.5%) (Fig 2). Other HIV-1 variants included C (n = 6, 6.9%), D (n = 19, 21.8%), G (n = 2, 2.3%), and Circulating Recombinant Forms (CRFs: n = 10, 11.5%) that comprised AD (n = 8, 9.2%) and A2D (n = 2, 2.3%).

### Nucleotide sequence accession numbers

All the 87 raw HIV-1 *pol* sequences reported in this article were deposited in the National Center for Biotechnology Information GenBank (https://www.ncbi.nlm.nih.gov/genbank/) and curated. Their accession numbers are KX790964 to KX791050.

### Factors associated with TDR

Only CD4 cell count was associated with the occurrence of TDR (p = 0.0145). Persons with TDR had a much lower CD4 median (cells/μL): 332 (IQR = 161.5–540.0) versus 589 (IQR = 420.5–726.0). No associations existed between TDR distribution and gender (p = 0.6750), participant’s age (p = 0.3068), acute vs recent HIV-1 infection (p = 1.0000), HIV-1 subtype (A: p = 1.0000, C: p = 0.4496, D: p = 1.0000, G: p = 1.0000, AD: p = 0.5535, A2D: p = 1.0000), and viral load (p = 0.8982).

## Discussion

We report the existence of a moderate level TDR in rural western Kenya, a level higher than the estimated prevalence (7.4%) in East Africa [7] and the overall weighted TDR prevalence (5.6%) in the high HIV prevalence sSA [27]. Global TDR estimates range from 8–22% [8–13,25,46]. The few independent studies in Kenya and East Africa have cited TDR prevalence of between 1–13% [16,26,27,32] and 0–19%, respectively [10,11,27–31,33]. Our results herein, including TDR prevalence, reflect information and activities as of 2012. Wide-scale and long duration of ARV use in this region could have contributed to the observed TDR prevalence [5,6]. Current ARV coverage among adults in this region is high, estimated at 50–69% [37] having been available for about nine years. Due to lack of temporal data especially after ARV scale-up [10], it remains unknown whether the 9.2% represents an increased TDR in this region. Published surveys have reported increasing TDR prevalence in Mombasa coastal Kenya: from 4.9% (2007/09) [27] to 13.2% (2009/10) [26]; Kampala central Uganda: from 7.0% (2002/04) [11] to 8.6% (2009/10) [29]; and Entebbe central Uganda: from 0.0% (2006/07) [33] to 19.2% (2006/09) [10], and decreasing TDR prevalence in Nairobi central Kenya: from 7.5% (2005) [32] to 4.5% (2007/09) [27]. The observed moderate TDR level in Ndhiwa is challenging to ART program due to increased risk of virological failure associated with pre-treatment drug resistance (PDR) [24]. The Stanford HIVdb revealed that about 9 of every 100 persons with AHI/RHI in Ndhiwa have viruses with mutations that confer at least potential low-level resistance to at least one of the ARV drug classes. Moreover, this together with the observed TDR have epidemiological consequences for further transmissions [14,22] owing to the high HIV incidence in this region [38].

Compared to other regions in sSA, the rates of TDR in East Africa have been shown to be increasing, with a projected annual increase of 29% since ART rollout [7]. Comparatively, the findings of this study showed a moderate level of TDR that was higher than that reported from most of the regions in East Africa [11,27–29,31–33] especially in studies from rural settings [16,28–31]. This can partly be explained by the high prevalence and incidence in the study region as well as the more accurate means for assessing incidence [37]. Among the studies compared, majority assessed TDR among persons initiating ART with unknown time of infection. Studies have shown possible archiving of TDR mutations due to comparatively higher viral fitness of the wild-type strains and it is likely that these studies may have underestimated the TDR levels as confounded by the choice of population studied [47]. The observed TDR in Ndhiwa was however lower than that observed in the urban settings of Mombasa (13.2%) and Entebbe (19.2%). The difference in this may be explained by the difference in the epidemic in the two regions. For example, in Mombasa HIV transmission occurs substantially through injection drug use (IDU) [48] while that in Ndhiwa it is mainly heterosexual. IDU has been associated with a higher risk of primary HIV transmission in many parts of the world [49]. On the other hand, ART rollout in Uganda and Kenya started in 2000 and 2003, respectively [27]. Thus, the length of ART use in Uganda is higher than that of Ndhiwa, which may partly explain the nearly 2 times TDR difference observed in the two regions, i.e., 19.2% in Entebbe (Uganda) against 9.2% in Ndhiwa [27].

As reported from other studies, NNRTI mutations comprised a majority of the detected TDRMs [10,16,17,25–27,29,30,32], with K103N/S predominating. The high occurrence of this mutation is due to its ease of transmission compared with PI- and NRTI-associated TDRMs [50] and the tendency to persist for long durations [12,51]. Moreover, the observed high level could be due to the previous use of nevirapine monotherapy or nevirapine tail in short-course triple ART PMTCT, which has been associated with increased risk of resistance [37,52]. Contrary to other studies in East Africa, we also observed a high frequency of M184V mutation [16,26,28,30–32]. The levels of observed NRTI mutations may suggest a change from the predominantly NNRTI to a combined RTI TDR epidemic, with majority of NRTI being M184V mutation. M184V is however generally highly revertant. Although studies have suggested a low fitness and hence transmission of M184V mutation, studies using more sensitive genotyping assays have shown that this mutation may still be transmitted [53] as minority variants. However, it is also likely that the observed mutations may be transmitted from patients on treatment who received non-standardized regimen or those failing treatment with resistance strains. Limited information exists pertaining acquired drug resistance from this population. Thus, more studies may be needed to verify whether the HIV TDR variants that we identified were transmitted from persons on HAART.

Phylogenetic analyses revealed that among persons with AHI/RHI, HIV-1 subtype A prevailed as the most frequent strain in circulation. HIV-1 subtypes C and G, and each of the variants of intersubtype recombinant mosaics (AD and A2D) were marginally present. Cumulatively, the frequency of CRFs was high. Some recombinants might indicate multiple infections [54] or independent transmission networks [55], but further analyses are essential to validate these. Consistent with previous molecular epidemiological assessments, we found subtypes A and D to be the most predominant strains that drive the epidemics in Kenya [16,26,32,56] and East Africa [11,28–31,33]. Intersubtype AD remained the most common HIV-1 recombinant [16,31,33]. Great HIV diversity can foil accurate diagnosis, effective treatment and vaccine design [57].

Low CD4 cell count was significantly associated with TDR, a plausible indication of a rapid disease progression associated with drug-resistant strains. TDR has been shown to have a profound impact on HIV-1 pathogenesis in recently infected individuals. Individuals infected with HIV-1 strains with TDR have a rapid depletion of CD4 cell count during the first year of infection [58]. The associations of TDR distribution with other variables were not statistically significant. Though one study reported an association between TDR prevalence and AHI (mean seroconversion period: 45 days) [12], in our study, we found no association of TDR with the AHI/RHI period. The absence of TDR mutations in persons with AHI in our survey could have resulted from missed minority-level archived mutations by DBS genotyping [59].

Shortcomings of the study included potential for misclassification (bias) associated with the serological assays for characterizing the age of infection. Thus, there is chance that a few chronic infections might have been misclassified as RHI. To minimize this, we used recent infection testing algorithm (RITA), based on two serological assays and surrogate markers to provide information on recency [18]. The two serological assays that we used have been observed to have a low false positive rate (FPR) in this population; BioRad (2.4) and LAg avidity (0.5%) [60]. This variation can be due to influence by HIV-1 subtype [61]. The low FPR (<2%) of LAg avidity was consistent with the characteristics of an acceptable assay [35]. Because RITA cannot eliminate all FPR, viral load measurements were included to reduce the FPR [18]. As compared to massive parallel sequencing, the population-based sequencing might have not detected “sentinel mutations” in quasispecies, thus, possibly underestimating the TDR in this population [4,62–64].

## Conclusions

Despite these limitations, we reveal the presence of a relatively higher TDR prevalence in rural western Kenya than in most regions within East Africa. We propose that the WHO-recommended programmatic actions for moderate TDR level be performed, including evaluation of early warning indicators and both regional and nationwide PDR survey to determine the causes of TDR and advise on choice of first-line treatment in case of persistent high-level PDR [34].

## Supporting information

S1 Fig MEGA phylogenetic maximum likelihood (ML) circular tree for HIV-1 subtyping of the partial *pol* gene from AHI/RHI antiretroviral-naïve persons in the Ndhiwa cohort. The evolutionary history was inferred from bootstrap phylogeny test (1000 replicates) using ML phylogenetic reconstruction based on Hasegawa-Kishino-Yano with Gamma distribution and Invariable sites (HKY+G+I) model. The tree was rooted on HIV-1 subtype K (marked with ●). The 19 reference sequences used are marked with ▼ beside their names. The clustering of the 87 viral sequences with respect to the reference sequences allowed for the identity of the associated taxa.
(TIF)
