# Supplementary figures and images for: Surveillance of HIV-1 pol transmitted drug resistance in acutely and recently infected antiretroviral drug-naïve persons in rural western Kenya
Source: PLoS One. 2017 Feb 8;12(2):e0171124. doi: 10.1371/journal.pone.0171124 (PMC5298248; doi:10.1371/journal.pone.0171124)

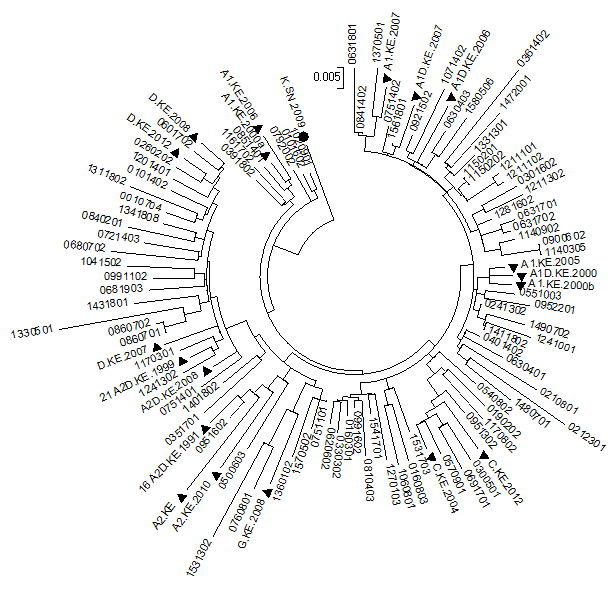

Supplement: S1 Fig — The evolutionary history was inferred from bootstrap phylogeny test (1000 replicates) using ML phylogenetic reconstruction based on Hasegawa-Kishino-Yano with Gamma distribution and Invariable sites (HKY+G+I) model. The tree was rooted on HIV-1 subtype K (marked with ●). The 19 reference sequences used are marked with ▼ beside their names. The clustering of the 87 viral sequences with respect to the reference sequences allowed for the identity of the associated taxa. (TIF) [file pone.0171124.s001.tif]
